# Perception about vaccines and level of knowledge, attitudes and practices towards COVID-19 in older adults in Lima, Peru

**DOI:** 10.17843/rpmesp.2022.392.10847

**Published:** 2022-06-30

**Authors:** Claudia L. Vidal-Cuéllar, Omar F. Zanoni-Ramos, Guiliana Mas, Tania Tello-Rodríguez

**Affiliations:** 1 Instituto de Gerontología, Universidad Peruana Cayetano Heredia, Lima, Peru. Universidad Peruana Cayetano Heredia Instituto de Gerontología Universidad Peruana Cayetano Heredia Lima Peru; 2 Facultad de Medicina, Universidad Peruana Cayetano Heredia, Lima, Peru. Universidad Peruana Cayetano Heredia Facultad de Medicina Universidad Peruana Cayetano Heredia Lima Peru; 3 Hospital Cayetano Heredia, Lima, Peru. Hospital Cayetano Heredia Lima Peru

**Keywords:** COVID-19, Aged, Health Knowledge, Attitudes, Practice, COVID-19 Vaccines

## Abstract

This study aimed to describe the perception about vaccines and the level of knowledge, attitudes and practices towards COVID-19 in older adults from a hospital in Lima, Peru. Descriptive and cross-sectional study carried out from March to November 2021. An instrument was adapted and validated to measure the level of knowledge, attitudes and practices; the perception about vaccines was evaluated with an exploratory questionnaire. Eighty-three older adults were surveyed, the mean age was 74.0 years and 62.7% were women. Most of the participants knew the cause and symptoms, and 50.6% believed that it could be transmitted by contaminated food. Additionally, 61.7% used traditional medicine to prevent it, and 65.4% considered that the level of social awareness was insufficient; 91.5% were vaccinated against COVID-19, and 65.4% considered these vaccines to be safe. In conclusion, most older adults showed a high level of knowledge, attitudes and practices and a positive perception about the vaccine against COVID-19.

## INTRODUCTION

By early 2022, the World Health Organization (WHO) reported approximately 300 million cases of COVID-19, more than five million deaths, and nine billion vaccines administered ^1^. COVID-19 has disproportionately affected older adults, highlighting their vulnerability as well as the frailty of health systems. Older adults are more likely to develop severe forms of the disease due to immune system changes and chronic diseases, which generate high rates of hospitalization and mortality ^2^. In 2021, Peru reported the highest per capita mortality rate for COVID-19 worldwide, attributed to the fragmentation of the health system and the scarcity of hospital resources ^3^ ^,^ ^4^. In Peru, older adults represent 13% of the national population ^5^ and 70% of deaths due to COVID-19 ^6^.

Vaccination is the most appropriate measure to control infection in the absence of effective therapeutic interventions. The vaccination process against COVID-19 in Peru began in February 2021, and by May 28, 2022, more than 70 million doses had been administered ^7^. During 2021, older adults were immunized with the Pfizer vaccine and in some regions with AstraZeneca. Delayed vaccination could spread variants that overcome the immunity conferred by previous vaccines or by the disease ^8^. Nevertheless, the low acceptance of vaccines is a problem that prevents the mitigation of the disease. According to surveys conducted in Peru, the acceptance of the COVID-19 vaccine reached 49-60% between January and March 2021 ^9^.

The level of knowledge about COVID-19 has an impact on taking preventive measures, so it is necessary to develop educational interventions for vulnerable populations ^10^. Worldwide, studies have been conducted to measure knowledge, attitudes and practices in various diseases through the KAP (Knowledge, Attitude and Practices) format; however, few studies have been conducted in older adults ^11^ ^,^ ^12^. Given the lack of evidence in Peru and considering the vulnerability of older adults during the current pandemic, the aim of this study was to describe the perception about vaccines and the level of knowledge, attitudes and practices related to COVID-19 in older adults from a general hospital in Lima, Peru.

**KEY MESSAGES**

Motivation for the study: The perception and level of knowledge, attitudes and practices towards COVID-19 influence the practice of preventive measures in society, which is important for older adults given their epidemiological vulnerability.

Main findings: Most of the older adults presented a high degree of acceptance towards vaccines against COVID-19 and an adequate level of knowledge, attitudes and practices.

Implications: It is important to provide education about COVID-19 to vulnerable populations, in order to prevent adverse health events. Studies in different regions of Peru are needed in order to implement specific educational interventions, given the sociocultural heterogeneity of the country.

## THE STUDY

### Study design

This was a descriptive, cross-sectional study.

### Population

The population consisted of 320 older adults aged 60 years and older who attended the Comprehensive Geriatric Assessment (VGI) clinic of the Cayetano Heredia Hospital from January 2019 to February 2020. The study included older adults aged 60 years and older who attended the VGI clinic and who had their own or a family member’s cell phone registered in the VGI clinic database. Quechua-speaking patients, and those with cognitive impairment, severe hearing loss or language disorder were excluded. We considered the total population was considered. Patients were evaluated in the physical, cognitive, affective, and social areas, and gave informed consent for subsequent telephone evaluations. 

### Instrument

The instrument in KAP format we used to assess knowledge, attitudes and practices towards COVID-19 was originally developed by the WHO based on the study by Erfani *et al*.^ (^ ^13^. This instrument was applied in Iran in those aged 15 years and older. It contains 53 multiple-choice questions. The instrument was culturally validated since it was originally written in English.

### Cultural validation

To validate the KAP instrument, the translation-retranslation method was used by three native Spanish-speaking bilingual physicians with an advanced level of English. This method consists of translating the instrument into the language of interest and then back-translating it into the original language ^14^. Simple matches between the translated sentences were searched for and converted into an average percentage. Then, the correlation of the percentage of matched sentences between translations was found, calculating a final average. Finally, the conceptual agreement between items was evaluated by simple reading. The same steps were carried out to evaluate the back-translations. The cultural validation process is described in the supplementary material (S1 and S2). 

The translated and back-translated version of the KAP instrument was submitted to a committee made up of two general practitioners and an internist, who prepared a consensus document (S3 and S4). With this validated version, we conducted a pilot test in the community on 58 older adults through social networks (S5 and S6). The final KAP instrument included 54 questions (S7). Perception about vaccines for COVID-19 was assessed with an exploratory questionnaire developed by the investigators (S8).

### Data Collection

The survey was conducted by telephone from March 24 to November 14, 2021. The survey lasted 20 min, the responses were recorded on data collection forms and then into a Microsoft Excel 2016 file.

The collected variables included sociodemographic characteristics (age, sex, marital status, years of education) and medical history (comorbidities and diagnosis of COVID-19 during the pandemic).

The variables on the perception about COVID-19 vaccines included vaccination history, number of doses, perceived safety of the vaccines, knowledge of their benefits, relevance of vaccination, and knowledge of preventive measures after vaccination. 

The variables of the KAP instrument were divided into:

Knowledge: evaluated the etiology, characteristics, form of presentation and management of the disease.

Attitudes: evaluated the perception of the severity of the disease, quarantine as a preventive measure, ways of disease transmission and acceptance of government measures.

Practices: evaluated isolation measures and prevention of contact with possible infected persons. As well as personal hygiene, perception of homeopathic medicine and source of information.

Statistical analysis 

Categorical variables are presented as frequencies and proportions; continuous variables are presented as mean and standard deviation (SD) or median and interquartile ranges (IQR), according to their normal distribution measured by the Shapiro-Wilk test.

Ethical Aspects

The ethical principles outlined in the Declaration of Helsinki were followed. The study was approved by the Ethics Committee of the Cayetano Heredia Hospital (CIE-HCH), code 124-2020.

## FINDINGS

A total of 320 older adults were contacted by telephone, of whom 220 did not answer the call, resulting in 100 who agreed to participate in the study. Of these, 17 were excluded because they did not meet the inclusion criteria. The final sample included 83 participants, 62.7% of whom were women. The mean age was 74.0 (SD: 7.7) years, and the most frequent marital status was married (46.9%). Of the participants, 25.3% reported having had COVID-19 during the pandemic (Table 1).

**Table 1:** Sociodemographic characteristics of the older adults in the study (n=83).

Variables	n (%)
Age ^a^	74.0 (7.7)
Sex	
Female	52 (62.7)
Male	31 (37.3)
Marital status	
Single	20 (24.1)
Married	39 (47.0)
Widower	16 (19.3)
Divorced	5 (6.1)
Cohabitant	3 (3.6)
Years of education	
0 years	9 (10.8)
1-6 years	39 (47.1)
7-11 years	27 (32.5)
12 years or more	8 (9.6)
COVID-19 diagnosis during the pandemic	21 (25.3)
Background	
Arterial hypertension	37 (44.6)
Diabetes *mellitus*	18 (21.7)
Heart failure	1 (1.2)
Chronic kidney disease	4 (4.8)
Arrythmia	3 (3.6)
Bronchial asthma	9 (10.8)
COPD	2 (2.4)
Hypothyroidism	6 (7.2)
Other	3 (3.7)

COPD: chronic obstructive pulmonary disease

a Mean (SD)

Regarding the perception about vaccines, 91.5% of the participants received at least one dose; 95% reported that the vaccines protect against severe forms of disease, 9.9% indicated that they are not safe (Table 2). Regarding knowledge, 98.8% have heard about the disease, 92.7% knew that it was contagious and 73.4% knew its viral etiology. The incubation period was unknown to 92.7% and 50.6% believed that it can be transmitted by dairy products or contaminated meat; 43.3% reported that there is no specific treatment or that it is only paracetamol, and 22.8% indicated that it is ivermectin (Table 3). Regarding attitudes, 96.3% considered it to be a serious disease and 65.4% reported that the level of social awareness of the disease was insufficient (Table 4). As for practices, 88.8% avoided leaving home to prevent infection, 93.8% avoided physical contact, 100% pay more attention to their hygiene and 61.7% used traditional medicine to prevent the disease (Tables 5).

**Table 2:** Perceptions about COVID-19 vaccines among older adults.

Item	n (%)
1. Have you had an influenza or pneumococcal vaccine? (n=81/83) ^a^	
Doesn’t know	3 (3.7)
No	20 (24.7)
Yes	58 (71.6)
2. If yes, which vaccine has been administered? (n=58)	
Pneumococcus	13 (22.4)
Influenza	9 (15.5)
Both	36 (62.1)
3. Have you been vaccinated against COVID-19? (n=71/83) ^a^	
Yes	65 (91.5)
No	6 (8.5)
4. If yes, indicate number of doses for COVID-19 (n=64/65) ^a^	
1st dose	6 (9.4)
2nd dose	58 (90.6)
5. Do you consider it to be the same influenza vaccine? (n=81/83) ^a^	
Doesn´t know	15 (18.5)
No	60 (74.1)
Yes	6 (7.4)
6. Do you think that a person not vaccinated against COVID-19 is likely to contract COVID-19 and could have complications, i.e. hospitalized and/or die? (n=80/83) ^a^	
Doesn´t know	7 (8.8)
No	1 (1.2)
Yes	72 (90.0)
7. Do you consider vaccines to be safe? (n=81/83)^ a^	
Doesn´t know	20 (24.7)
No	8 (9.9)
Yes	53 (65.4)
8. Do you think vaccines can prevent severe forms of disease? (n=80/83) ^a^	
Doesn´t know	3 (3.8)
No	1 (1.2)
Yes	76 (95.0)

a Missing data

**Table 3:** COVID-19 knowledge among older adults.

Item	n (%)
K1. I have heard about COVID-19. (Answer: yes)	82 (98.8)
K2. COVID-19 is a contagious disease. (Answer: yes)	77 (92.7)
K3. Which of the following is a cause of COVID-19? (Answer: virus)	61 (73.4)
K4. What is the length of the incubation period of the disease? (Answer: don't know)	77 (92.7)
K5. Which of the following is the treatment for COVID-19? (Answer: no treatment/paracetamol) ^a^	36 (43.3)
K6. In which age group is the disease most dangerous? (Answer over 50 years old) ^b^	38 (45.7)
K7. Fever is a symptom of COVID-19. (Answer: yes)	67 (80.7)
K8. Coughing is a symptom of COVID-19. (Answer: yes)	65 (92.7)
K9. A sore throat is a symptom of COVID-19. (Answer: yes)	71 (85.5)
K10. Body pain is a symptom of COVID-19. (Answer: yes)	72 (86.7)
K11. Diarrhea or constipation is a symptom of COVID-19. (Answer: yes)	66 (79.5)
K12. Headache is a symptom of COVID-19. (Answer: yes)	70 (84.3)
K13. If I suspect a COVID-19 infection, first of all, I measure the fever. (Answer: yes)	69 (84.1)
K14. If I suspect a COVID-19 infection, first of all, I seek a physician. (Answer: yes)	72 (87.8)
K15. If I suspect a COVID-19 infection, I avoid unnecessary daily activities. (Answer: yes)	73 (89.0)
K16. To avoid contracting COVID-19, I avoid contact with individuals suspected of being infected with COVID-19. (Answer: yes)	78 (95.1)
K17. The number of cases of the disease is increasing in Peru. (Answer: yes)	44 (54.3)
K18. Hand washing with soap and water can eliminate the cause of the disease. (Answer: yes)	75 (92.5)
K19. The disease can be transmitted directly by coughing. (Answer: yes)	72 (88.8)
K20. The disease can be transmitted directly through contact with infected surfaces. (Answer: yes)	64 (79.0)
K21. The disease can be transmitted directly through the consumption of contaminated dairy products and meat. (Answer: yes)	41 (50.6)
K22. The disease can be transmitted directly through contact with infected individuals. (Answer: yes)	77 (95.0)
K23. The disease is more dangerous in pregnant women. (Answer: yes)	58 (71.6)
K24. The disease is more dangerous in older individuals. (Answer: yes)	78 (96.3)
K25. The disease is more dangerous in people with weakened immune systems. (Answer: yes)	78 (96.3)
K26. The disease is more dangerous in people with cancer, diabetes and chronic respiratory diseases. (Answer: yes)	77 (95.0)

a Alternatives: a) Paracetamol / No treatment, b) Ivermectin, c) Antibiotics, d) Others (anticoagulants, corticosteroids, herbs), e) Doesn't know

b Alternatives: a) Under 15 years old, b) 15 to 30 years old, c) 30 to 50 years old, d) Over 50 years old, e) All ages, f) Doesn't know

**Table 4:** Attitudes about COVID-19 in older adults.

Item	n (%)
A1. Do you believe that early detection of COVID-19 can improve treatment and outcome? (Answer: yes)	73 (90.1)
A2. I believe that COVID-19 can be treated at home. (Answer: yes)	69 (85.1)
A3. I believe that health education can prevent COVID-19. (Answer: yes)	79 (97.5)
A4. I believe that COVID-19 is a severe disease. (Answer: yes)	78 (96.3)
A5. I believe that COVID-19 can be prevented by proper quarantine. (Answer: yes)	71 (87.6)
A6. I believe that, if a vaccine is available for the disease, it should be used. (Answer: yes)	77 (95.0)
A7. I believe that COVID-19 is a curable disease. (Answer: yes)	66 (81.4)
A8. I believe that the level of awareness of society about COVID-19 is sufficient. (Answer: no)	53 (65.4)
A9. I believe that COVID-19 causes death in all cases. (Answer: no)	57 (70.3)
A10. I believe that COVID-19 can be transmitted to humans through pets. (Answer: no)	34 (41.9)
A11. I believe that authorities should restrict travel to and from COVID-19 areas to prevent contamination. (Answer: yes)	74 (91.3)
A12. I believe that the authorities should quarantine patients with COVID-19 in special hospitals. (Answer: yes)	69 (85.1)
A13. I believe that due to the increase in the number of COVID-19 cases, they were right to close the educational institutions. (Answer: yes)	77 (95.0)
A14. I believe that due to the increase in the number of COVID-19 cases, they have done well to close religious places such as churches. (Answer: yes)	68 (83.9)
A15. I believe that because of the increase in the number of COVID-19 cases, the authorities should be ready to close and quarantine the city again. (Answer: yes)	54 (66.6)

**Table 5:** COVID-19 practices in older adults.

Item	n (%)
P1. In order to prevent the contagion and spread of COVID-19 I avoid leaving my home. (Answer: yes)	72 (88.8)
P2. In order to prevent the spread of COVID-19, I avoid taking unnecessary trips. (Answer: yes)	76 (93.8)
P3. In order to prevent the transmission and spread of COVID-19 I avoid eating food away from home. (Answer: yes)	79 (97.5)
P4. In order to prevent the spread and spread of COVID-19, I avoid shaking hands, hugging and kissing. (Answer: yes)	76 (93.8)
P5. In order to prevent the transmission and spread of COVID-19, I wash my hands frequently. (Answer: yes)	81 (100.0)
P6. In order to prevent contagion and spread of COVID-19, I pay more attention than usual to my personal hygiene. (Answer: yes)	81 (100.0)
P7. In order to prevent the spread of COVID-19, I use disinfectants and solutions (bleach, alcohol gel). (Answer: yes)	75 (92.5)
P8. In order to prevent the spread of COVID-19, I use herbal products and traditional medicine. (Answer: yes)	50 (61.7)
P9. In order to prevent the spread of COVID-19, I take vitamin supplements. (Answer: no)	54 (66.6)
P10. In order to prevent the transmission and spread of COVID-19, when do you wear face masks? (Answer: public places) ^a^	40 (49.3)
P11. With the intention of preventing the spread of COVID-19, do you think that (gargle, eucalyptus, garlic or ginger) can prevent it? (Answer: yes)	50 (62.5)
P12. With the intention of curing or treating COVID-19 do you think that (gargle, eucalyptus, garlic or ginger) can cure it? (Answer: no)	43 (53.7)
P13. What is your main source of information about knowledge of the disease? (Answer: television) ^b^	29 (35.8)

a Alternatives: a) Never, b) Public and crowded places, c) Most of the time, d) Always, e) Don't know, f) Doesn't know.

b Alternatives: a) Television, b) Radio, c) Newspapers, d) Social networks, e) Health care personnel, f) Family and friends, g) Neighbors

## DISCUSSION

In our study, most of the older adults recognized vaccines as a preventive measure, and also showed an adequate level of knowledge, attitudes and practices towards COVID-19. However, we also found erroneous beliefs that could influence their preventive behaviors in the face of the pandemic.

Regarding the perception about COVID-19 vaccines, most participants reported having been vaccinated and receiving two doses, which is similar to the local epidemiological situation during the study period (96.6% of older adults vaccinated with a second dose in northern Lima and 90% at the national level) ^7^. These findings suggest that vaccine acceptance in this age group is high. This contrasts with a study conducted at the beginning of vaccination in Peru, where the intention to vaccinate in older adults was 64.4% ^15^. The difference could be explained by to the massive vaccination campaigns carried out at the national level; however, vaccination could also have been influenced by the decision of the older adult’s family members.

Regarding knowledge, almost all participants are aware of the viral etiology and symptoms. This could be attributed to the fact that the Ministry of Health promoted the dissemination of information through the media since the beginning of the pandemic. These findings differ from international studies in which the level of knowledge is inversely related to age ^12^ ^,^ ^16^. On the other hand, participants reported erroneous beliefs about the natural history of the disease, such as the possibility of foodborne transmission. In addition, most were unaware of the incubation period, suggesting that they may have been unaware of the isolation time required after exposure to a contact or a person diagnosed with COVID-19. Regarding treatment, responses were not uniform, which could be explained by the changing measures adopted to deal with the pandemic in Peru. For example, the Peruvian government promoted the use of ivermectin, hydroxychloroquine and azithromycin at the beginning of the pandemic, which could explain the heterogeneity of the responses.

Another relevant finding was that almost half of the participants considered the disease to be more dangerous in adults older than 50 years; a similar proportion stated that it affects all ages equally. This shows that a significant proportion underestimates the risk of older adults to have severe forms of COVID-19. However, it is important to consider that during the study period there was an increase in cases in young adults in our country, which could have influenced the participants’ perspective. Several international studies have shown that older adults’ risk perception about COVID-19 is underestimated, which is attributed to a phenomenon of paradoxical illusory optimism ^17^.

Regarding attitudes, most older adults recognized that COVID-19 is a serious disease, and that health education and quarantine can prevent its transmission. A notable finding in this domain was that more than half of the participants felt that society does not have sufficient awareness of the impact of COVID-19. In terms of practices against this disease, most adopted biosecurity measures to avoid contagion, as described in other studies ^12^ ^,^ ^17^. In addition, more than half used herbal products and traditional medicine to prevent infection. Similarly, a study in an HIV-infected Peruvian population found that almost half of the participants (46.8%) believed that gargling with salt and water prevents COVID-19 ^18^. Another study in Cusco showed that 80.2% used medicinal plants for prevention, while 71% used them to treat COVID-19 ^19^.

A strength of our study is that it is one of the first to explore the perception of older adults about COVID-19 vaccines and the pandemic in Peru. The main limitations were the small sample size due to the limited use of cell phones among older adults. In this study, only 10% of respondents had their own cell phone, which may be due to low economic resources. In addition, the constant change of cell phone numbers in the family may have limited contact with the older adult, which would explain the poor response rate in the study.

On the other hand, during the study period, the number of COVID-19 cases was decreasing due to vaccination, which is evident from the heterogeneity of the participants’ responses. For example, in mid-April 2021, there were about 1600 cases per day, whereas in September 2021, there were about 1000 cases per day ^20^. In addition, the use of the telephone was not appropriate for data collection, given the length of the survey. Also, participants’ responses may have been influenced by family members who were present during the call. Because the participants were all from the city of Lima, the results cannot be extrapolated to other regions of Peru. Also, the cultural validation of the instrument cannot be applied to populations with mother tongues other than Spanish.

In conclusion, most of the older adults surveyed had a positive perception of the COVID-19 vaccine and an acceptable level of knowledge, attitudes and practices towards this disease. However, they also presented numerous erroneous beliefs that could have an impact on preventive practices. Further studies in older adults are needed to obtain evidence on their perceptions and thus implement health and educational strategies in the face of possible future pandemics.
