# Supplementary material for: Perception about vaccines and level of knowledge, attitudes and practices towards COVID-19 in older adults in Lima, Peru
Source: Rev Peru Med Exp Salud Publica. 2022 Jun 30;39(2):201–7. doi: 10.17843/rpmesp.2022.392.10847 (PMC11397665; doi:10.17843/rpmesp.2022.392.10847)
Supplement: Supplementary material. — Available in the electronic version of the RPMESP. [file rpmesp-39-02-10847-s001.docx]

**MATERIAL SUPLEMENTARIO**

**S1. Adaptación cultural del instrumento KAP**

Se procedió a la validación cultural del cuestionario KAP. En la **figura S3** se puede apreciar un flujograma de la metodología usada. Las traducciones y retrotraducciones estuvieron a cargo de tres médicos bilingües (dos médicos generales y un médico internista) con lengua materna el español y nivel de inglés avanzado con categoría internacional C1. Cada uno de ellos tradujo y retrotradujo el cuestionario por separado. La evaluación de las traducciones del inglés al español consistió en: primero, hallar las coincidencias simples de las frases y convertirlas en porcentajes entre las traducciones del médico-lingüista 1 (ML1) y médico-lingüista 2 (ML2), médico-lingüista 1 (ML1) y médico-lingüista 3 (ML3), médico-lingüista 2 (ML2) y médico-lingüista 3 (ML3); de estos valores se obtendrá un promedio.

Segundo, se halló la correlación del porcentaje de las frases coincidentes entre las traducciones efectuadas por los expertos, hallándose también un promedio final de estos porcentajes. Si las variables tenían distribución normal se realizó correlación de Pearson, sino se realizaba correlación de Spearman (se consideró como estadísticamente significativo un valor de p<0,05). Tercero; se efectuó una lectura simple de cada ítem de las traducciones y se obtuvo el significado conceptual de la traducción, en dos opciones; si concuerda y no concuerda conceptualmente.

Para evaluar las concordancias entre las variables categóricas se obtuvo el índice de Kappa. La evaluación de las retrotraducciones del español al inglés consistió en: primero, hallar las coincidencias simples de las frases, y convertirlas en porcentajes, entre las retro traducciones del ML1 y ML2, ML1 y ML3, ML2 y ML3; de estos valores se obtuvo un promedio. Luego, se halló la correlación del porcentaje de las frases coincidentes entre las retrotraducciones efectuadas por los expertos, hallándose así un promedio final de estos porcentajes de las retrotraducciones. Si las variables tenían distribución normal se realizó correlación de Pearson, sino se realizaba correlación de Spearman (se consideró como estadísticamente significativo un valor de p<0,05. Tercero, se efectuó una lectura simple de cada ítem de las retraducciones y se obtuvo el significado conceptual de la re traducción, en dos opciones: si concuerda y no concuerda conceptualmente. Para evaluar las concordancias entre las variables categóricas se obtuvo el índice de Kappa. Para el análisis los datos se transfirieron a un *software* estadístico STATA versión 16.

Posteriormente, mediante un comité de cuatro expertos revisaron, analizaron e identificaron preguntas incongruentes y otras preguntas pertinentes a nuestro medio social-cultural en el momento de la pandemia. Por tal motivo se agregaron tres preguntas en el dominio de prácticas quedando como documento final (ver S8). En el tiempo en el que se inició el desarrollo del proyecto coincidió con el inicio de la vacunación a la población adulta mayor, motivo por el cual a sugerencia de los expertos en el contexto dinámico en el que nos encontrábamos se agregaron preguntas respecto a este tema.

En los resultados de la evaluación de la concordancia entre las traducciones se mostraron diferentes grados de acuerdo en los tres grupos. La concordancia entre las retrotraducciones (castellano-inglés) en los dominios de conocimientos y prácticas del (ML1-ML2), (ML1-ML3) y (ML2-ML3) fue alta (*Kappa*:1) (ver S4)**.** La correlación por grupo de traducciones mostró una correlación significativa entre ML1-ML3. Al evaluar la correlación por grupos de las retrotraducciones fue significativa en el dominio de conocimientos. Al emparejar ML1-ML3 no hubo correlación en el dominio de prácticas (r: 0,516, p=0,08) y sí el dominio en el de actitudes (r: 0,625, p=0,01) (ver S5).

**S2.** Flujograma del proceso de validación cultural


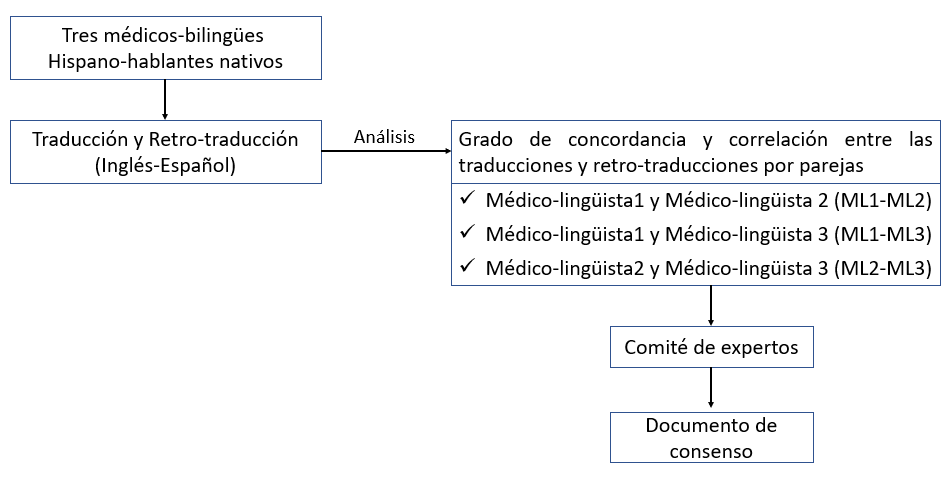


**S3.** Grado de acuerdo entre grupo de traducciones y retrotraducciones

| **Evaluadores** | **Acuerdo observado** | **Acuerdo esperado** | ***Kappa*** | **p** |
| --- | --- | --- | --- | --- |
| **Dominio Conocimientos** | | | | |
| *Traducción del inglés al castellano* |  |  |  |  |
| Médico-Bilingüe 1vs Médico-Bilingüe 2 | 96,5% | 96,5% | 0,00 | 0,5000 |
| Médico-Bilingüe 1 vs Médico-Bilingüe 3 | 100% | 92,6% | 1 | 0,0000 |
| Médico-Bilingüe 2 vs Médico-Bilingüe 3 | 96,5% | 96,5% | 0,00 | 0,5000 |
| *Retraducciones del castellano al inglés* |  |  |  |  |
| Médico-Bilingüe 1vs Médico-Bilingüe 2 | 100% | 100% | 1 | - |
| Médico-Bilingüe 1 vs Médico-Bilingüe 3 | 100% | 100% | 1 | - |
| Médico-Bilingüe 2 vs Médico-Bilingüe 3 | 100% | 100% | 1 | - |
| **Dominio Actitud** | | | | |
| *Traducción del inglés al castellano* |  |  |  |  |
| Médico-Bilingüe 1vs Médico-Bilingüe 2 | 86,67% | 86,67% | 0,000 | 0,5000 |
| Médico-Bilingüe 1 vs Médico-Bilingüe 3 | 100% | 76,89% | 1 | 0,0001 |
| Médico-Bilingüe 2 vs Médico-Bilingüe 3 | 86,67% | 86,67% | 0,000 | 0,5000 |
| *Retraducciones del castellano al inglés* |  |  |  |  |
| Médico-Bilingüe 1vs Médico-Bilingüe 2 | 86,67% | 86,67% | 0,000 | 0,5000 |
| Médico-Bilingüe 1 vs Médico-Bilingüe 3 | 100% | 76,89% | 1 | 0,0001 |
| Médico-Bilingüe 2 vs Médico-Bilingüe 3 | 86,67% | 86,67% | 0,000 | 0,5000 |
| **Dominio Prácticas** | | | | |
| *Traducción del inglés al castellano* |  |  |  |  |
| Médico-Bilingüe 1vs Médico-Bilingüe 2 | 100% | 84,72% | 1 | 0,0003 |
| Médico-Bilingüe 1 vs Médico-Bilingüe 3 | 100% | 84,71% | 1 | 0,0003 |
| Médico-Bilingüe 2 vs Médico-Bilingüe 3 | 100% | 84,71% | 1 | 0,0003 |
| *Retraducciones del castellano al inglés* |  |  |  |  |
| Médico-Bilingüe 1vs Médico-Bilingüe 2 | 100% | 100% | 1 | - |
| Médico-Bilingüe 1 vs Médico-Bilingüe 3 | 100% | 100% | 1 | - |
| Médico-Bilingüe 2 vs Médico-Bilingüe 3 | 100% | 100% | 1 | - |

**S4.** Correlación entre los grupos de traducciones y retraducciones

| **Evaluadores** | **r** | **p** |
| --- | --- | --- |
| **Dominio Conocimientos** | | |
| *Traducción del inglés al castellano* |  |  |
| Médico Bilingüe 1vs Médico-Bilingüe 2 | 0,208 | 0,305 |
| Médico Bilingüe 1 vs Médico-Bilingüe 3 | 0,580 | 0,001 |
| Médico Bilingüe 2 vs Médico-Bilingüe 3 | 0,350 | 0,070 |
| *Retraducciones del castellano al inglés* |  |  |
| Médico Bilingüe 1vs Médico-Bilingüe 2 | 0,884 | 0,000 |
| Médico Bilingüe 1 vs Médico-Bilingüe 3 | 0,725 | 0,000 |
| Médico Bilingüe 2 vs Médico-Bilingüe 3 | 0,682 | 0,000 |
| **Dominio Actitud** | | |
| *Traducción del inglés al castellano* |  |  |
| Médico Bilingüe 1vs Médico-Bilingüe 2 | 0,725 | 0,000 |
| Médico Bilingüe 1 vs Médico-Bilingüe 3 | 0,798 | 0,000 |
| Médico Bilingüe 2 vs Médico-Bilingüe 3 | 0,080 | 0,775 |
| *Retraducciones del castellano al inglés* |  |  |
| Médico Bilingüe 1vs Médico-Bilingüe 2 | -0,005 | 0,985 |
| Médico Bilingüe 1 vs Médico-Bilingüe 3 | 0,625 | 0,012 |
| Médico Bilingüe 2 vs Médico-Bilingüe 3 | -0,366 | 0,178 |
| **Dominio Prácticas** | | |
| *Traducción del inglés al castellano* |  |  |
| Médico Bilingüe 1vs Médico-Bilingüe 2 | 0,416 | 0,178 |
| Médico Bilingüe 1 vs Médico-Bilingüe 3 | 0,880 | 0,000 |
| Médico Bilingüe 2 vs Médico-Bilingüe 3 | 0,424 | 0,169 |
| *Retraducciones del castellano al inglés* |  |  |
| Médico Bilingüe 1vs Médico-Bilingüe 2 | 0,909 | 0,00 |
| Médico Bilingüe 1 vs Médico-Bilingüe 3 | 0,516 | 0,08 |
| Médico Bilingüe 2 vs Médico-Bilingüe 3 | 0,591 | 0,04 |

r: correlación Spearman o Pearson
p: valor p

**S5.** Desarrollo del estudio piloto

Se realizó un estudio piloto con el objetivo de realizar la validación del documento consenso. Se calculó que para un nivel de confianza del 95% con una probabilidad de identificar el problema en 0,05 incluyó a 58,5 participantes (*Viechtbauer W, Smits L, Kotz D, Budé L, Spigt M, Serroyen J, Crutzen R, A simple formula for the calculation of sample size in pilot studies, Journal of Clinical Epidemiology (2015), doi: 10.1016/j.jclinepi.2015.04.014*). El documento se elaboró en formato Google Forms y se envió por medio de redes sociales (Facebook, WhatsApp, etc.) a través de muestreo no probabilístico de bola de nieve de adultos mayores de 60 años a más que usarán las redes mencionadas. Se lograron captar a 58 participantes, de los cuales 62,07% fueron mujeres y 37,93% varones, con una edad de mediana 67 (62-74) años. La **tabla S7** muestra los valores de Alpha de Cronbach de cada uno de los dominios. El dominio de conocimiento se encuentra dentro de los niveles de fiabilidad regular, asimismo, la fiabilidad de los dominios de actitudes y prácticas fue regular-buena.

**S6.** Valores de Alpha de Cronbach de cada dominio

| **Dominio** | **Valor de Alpha de Cronbach** |
| --- | --- |
| Dominio de conocimientos | 0,30 |
| Dominio de actitudes | 0,45 |
| Dominio de prácticas | 0,42 |

**S7.** Instrumento para evaluar conocimientos, actitudes y prácticas relacionados con la COVID-19

| **Conocimiento** |
| --- |
| K1. Usted ha escuchado acerca de la COVID-19.  Sí ( ) No ( ) No sabe ( )  K2. La COVID-19 es una enfermedad contagiosa.  Sí ( ) No ( ) No sabe ( )  K3. ¿La COVID-19 es causado por? Marcar con una X   - Bacteria ( ) - Virus ( ) - Parásito ( ) - No sabe ( )   K4. ¿Ud. Conoce cuántos días demora desde que el virus de la COVID 19 ingresa a su organismo y empieza a producir los síntomas de tos, fiebre, dolor de espalda, dolor de garganta? Marcar con X   - Menos de 2 días ( ) - Entre 2 y 5 días ( ) - Entre 3 y 14 días ( ) - No sabe ( )   K5. ¿Cuál de los siguientes es el tratamiento para la COVID-19?  – Anticoagulantes ( ) - Antibióticos ( ) - Ivermectina ( ) - Corticoides ( )  – Paracetamol ( ) - No hay tratamiento ( )  K6. ¿En cuál grupo de edades la enfermedad es más peligrosa?  - Menores de 15 años ( ) -Entre 15 y 30 años ( ) -Entre 30 y 50 años ( )  - Mayores de 50 años ( ) - Mayores de 60 años ( ) - En todas las edades ( ) - No sabe ( )  K7. La fiebre es un síntoma de la COVID-19.  Sí ( ) No ( ) No sabe ( )  K8. La tos es un síntoma de la COVID-19.  Sí ( ) No ( ) No sabe ( )  K9. El dolor de garganta es un síntoma de la COVID-19.  Sí ( ) No ( ) No sabe ( )  K10. El dolor de cuerpo es un síntoma de la COVID-19.  Sí ( ) No ( ) No sabe ( )  K11. La diarrea puede ser un síntoma de la COVID-19.  Sí ( ) No ( ) No sabe ( )  K12. El dolor de cabeza es un síntoma de la COVID-19.  Sí ( ) No ( ) No sabe ( )  K13. Si usted sospecha que tiene COVID-19, antes que todo se toma la temperatura.  Sí ( ) No ( ) No sabe ( )  K14. Si sospecho de una infección por COVID-19, antes que todo, busco a un médico.  Sí ( ) No ( ) No sabe ( )  K15. Si sospecho de una infección por COVID-19, evito realizar actividades diarias innecesarias.  Sí ( ) No ( ) No sabe ( )  K16. Para evitar contraer la COVID-19, yo evito el contacto con individuos con sospecha de estar infectados por COVID-19.  Sí ( ) No ( ) No sabe ( )  K17. El número de casos de la enfermedad de la COVID-19 está aumentando en Perú.  Sí ( ) No ( ) No sabe ( )  K18. Lavarse las manos con agua y jabón puede eliminar la causa de la enfermedad.  Sí ( ) No ( ) No sabe ( )  K19. La enfermedad puede ser transmitida directamente mediante la tos.  Sí ( ) No ( ) No sabe ( )  K20. La enfermedad puede ser transmitida directamente mediante el contacto con superficies infectadas.  Sí ( ) No ( ) No sabe ( )  K21. La enfermedad puede ser transmitida directamente mediante el consumo de lácteos y carne contaminados.  Sí ( ) No ( ) No sabe ( )  K22. La enfermedad puede ser transmitida directamente mediante el contacto con individuos infectados (por el saludo de manos, abrazos, besos).  Sí ( ) No ( ) No sabe ( )  K23. La enfermedad es más peligrosa en mujeres gestantes.  Sí ( ) No ( ) No sabe ( )  K24. La enfermedad es más peligrosa en personas adultos mayores.  Sí ( ) No ( ) No sabe ( )  K25. La enfermedad es más peligrosa en personas con sistemas inmunes debilitados.  Sí ( ) No ( ) No sabe ( )  K26. La enfermedad es más peligrosa en personas con cáncer, diabetes y enfermedades respiratorias crónicas.  Sí ( ) No ( ) No sabe ( ) |
| **Actitudes** |
| A1. Usted cree que la detección temprana de la COVID-19 puede mejorar el tratamiento y el desenlace final.  Sí ( ) No ( ) No sabe ( )  A2. Usted cree que la COVID-19 puede ser tratado en casa.  Sí ( ) No ( ) No sabe ( )  A3. Usted cree que la educación en salud (medidas preventivas) puede prevenir la COVID-19.  Sí ( ) No ( ) No sabe ( )  A4. Usted cree que la COVID-19 es una enfermedad grave.  Sí ( ) No ( ) No sabe ( )  A5. Usted cree que la COVID-19 puede ser prevenido mediante una adecuada cuarentena.  Sí ( ) No ( ) No sabe ( )  A6. Usted cree que las vacunas disponibles para la COVID-19 deben de usarse.  Sí ( ) No ( ) No sabe ( )  A7. Usted cree que la COVID-19 es una enfermedad curable.  Sí ( ) No ( ) No sabe ( )  A8. Usted cree que nuestra sociedad tiene conocimiento de la magnitud que está causando la COVID-19.  Sí ( ) No ( ) No sabe ( )  A9. Usted cree que la COVID-19 produce la muerte en todos los casos.  Sí ( ) No ( ) No sabe ( )  A10. Usted cree que la COVID-19 puede ser transmitido a los humanos a través de las mascotas.  Sí ( ) No ( ) No sabe ( )  A11. Usted cree que las autoridades deben restringir los viajes desde y hacia las áreas con COVID-19 para prevenir los contagios  Sí ( ) No ( ) No sabe ( )  A12. Usted cree que las autoridades deben poner en cuarentena a los pacientes con COVID-19 en hospitales especiales.  Sí ( ) No ( ) No sabe ( )  A13. Usted cree que por el incremento en el número de casos de COVID-19 han hecho bien en cerrar los centros educativos.  Sí ( ) No ( ) No sabe ( )  A14. Usted cree que por el incremento en el número de casos de la enfermedad COVID-19 han hecho bien en cerrar lugares religiosos como las iglesias.  Sí ( ) No ( ) No sabe ( )  A15. Usted cree que por el incremento del número de casos de COVID-19 las autoridades deben estar listas para cerrar y poner nuevamente en cuarentena a la ciudad.  Sí ( ) No ( ) No sabe ( ) |
| **Prácticas** |
| P1. Con la intención de prevenir el contagio y la transmisión de la COVID-19, usted evita salir de su casa.  Sí ( ) No ( ) No sabe ( )  P2. Con la intención de prevenir el contagio y la transmisión de la COVID-19, usted evita tomar viajes innecesarios.  Sí ( ) No ( ) No sabe ( )  P3. Con la intención de prevenir el contagio y la transmisión de la COVID-19, usted evita comer comida fuera de casa.  Sí ( ) No ( ) No sabe ( )  P4. Con la intención de prevenir el contagio y la transmisión de la COVID-19, usted evita saludar con la mano, abrazar y besar.  Sí ( ) No ( ) No sabe ( )  P5. Con la intención de prevenir el contagio y la transmisión de la COVID-19, usted evita ir a trabajar.  Sí ( ) No ( ) No sabe ( )  P6. Con la intención de prevenir el contagio y la transmisión de la COVID-19, usted se lava las manos frecuentemente.  Sí ( ) No ( ) No sabe ( )  P7. Con la intención de prevenir el contagio y la transmisión de la COVID-19, usted le presta más atención que la usual a su higiene personal.  Sí ( ) No ( ) No sabe ( )  P8. Con la intención de prevenir el contagio y la transmisión de la COVID-19, usted usa desinfectantes y soluciones (lejía, alcohol en gel).  Sí ( ) No ( ) No sabe ( )  P9. Con la intención de prevenir el contagio de la COVID-19, usted utiliza productos a base de hierbas y medicina tradicional.  Sí ( ) No ( ) No sabe ( )  P10. Con la intención de prevenir el contagio de la COVID-19, ¿usted toma suplementos vitamínicos?  Sí ( ) No ( ) No sabe ( )  Si la respuesta es Sí, indicar cuales: vitamina D ( ), zinc ( ),  P11. Con la intención de prevenir el contagio y la transmisión de la COVID-19, ¿cuándo usa usted su mascarilla?  - Nunca ( ) - Solo en lugares públicos y llenos de gente ( )  - La mayoría del tiempo ( ) - Siempre ( ) - No sabe no opina ( )  P12. Con la intención de prevenir el contagio de la covid-19, ¿usted piensa que las gárgaras, el eucalipto, los ajos, o el kion pueden prevenirlo?  Sí ( ) No ( ) No sabe ( )  P13. Con la intención de curar o tratar la covid-19, ¿usted piensa que las gárgaras, el eucalipto, los ajos o el kion pueden curarlo?  Si ( ) No ( ) No sabe ( )  P14. ¿Cuál es su principal fuente de información sobre el conocimiento de la enfermedad por COVID 19? Marcar varias respuestas  - Televisión ( ) -Radio ( ) -Periódicos ( ) - Redes sociales (Facebook, Instagram) ( )  - Personal de salud ( ) - Familia y amigos ( ) - Vecinos ( ) - Grupos religiosos ( )  - Comedores populares ( ) |

**S8.** Instrumento sobre percepción de vacunas contra COVID-19

| **Percepción sobre vacunas contra COVID-19** |
| --- |
| 1. ¿Se ha puesto la vacuna contra el Neumococo (única dosis) y/o la influenza (todos los años)? 2. Sí 3. No 4. No sabe. 5. Si la respuesta fue afirmativa, especificar cuál: 6. Neumococo 7. Influenza 8. Ambas 9. ¿Usted ha sido vacunado para la COVID -19? 10. Sí 11. No 12. Si la respuesta fue afirmativa, especificar número de dosis 13. 01 dosis 14. 02 dosis 15. ¿Considera que la vacuna contra la COVID-19 es la misma que contra la influenza? 16. Sí 17. No 18. No sabe 19. ¿Cree que una persona **no vacunada** contra COVID-19 probablemente contraerá COVID-19 y que podría complicarse, es decir, hospitalizarse y/ o morir? 20. Sí 21. No 22. No sabe 23. ¿Considera que las vacunas de COVID-19 son seguras? 24. Sí 25. No 26. No sabe 27. ¿Sabe que las vacunas para la COVID-19 son una buena manera de proteger su salud y de evitar las formas severas de la enfermedad (hospitalización y muerte)? 28. Sí 29. No 30. No sabe |
