# Supplementary material for: Perception about vaccines and level of knowledge, attitudes and practices towards COVID-19 in older adults in Lima, Peru
Source: Rev Peru Med Exp Salud Publica. 2022 Jun 30;39(2):201–7. doi: 10.17843/rpmesp.2022.392.10847 (PMC11397665; doi:10.17843/rpmesp.2022.392.10847)
Supplement: Translation

# Translation: Percepción sobre las vacunas y nivel de conocimientos, actitudes y prácticas hacia la COVID-19 en adultos mayores de Lima, Perú

**Authors:** Claudia L. Vidal-Cuéllar, Omar F. Zanoni-Ramos, Guiliana Mas, Tania Tello-Rodríguez

**Affiliations:** 1 Instituto de Gerontología, Universidad Peruana Cayetano Heredia, Lima, Perú.; 2 Facultad de Medicina, Universidad Peruana Cayetano Heredia, Lima, Perú.; 3 Hospital Cayetano Heredia, Lima, Perú.

**Keywords:** COVID-19, Adulto mayor, Conocimientos, Actitudes y Práctica en Salud, Vacunas contra la COVID-19

## Abstract

El objetivo fue describir la percepción sobre las vacunas y el nivel de conocimientos, actitudes y prácticas hacia la COVID-19 en adultos mayores que acuden a un hospital en Lima, Perú. Estudio descriptivo y transversal de marzo a noviembre del 2021. Se adaptó y validó un instrumento para medir el nivel de conocimientos, actitudes y prácticas, y se evaluó la percepción sobre vacunas con un cuestionario exploratorio. Se encuestaron a 83 adultos mayores, la media de edad fue 74,0 años y el 62,7% fueron mujeres. La mayoría conoce la causa y síntomas, y el 50,6% cree que puede transmitirse por alimentos contaminados. El 61,7% usa medicina tradicional para prevenirla, y el 65,4% considera insuficiente el nivel de conciencia social. El 91,5% se vacunó contra la COVID-19, y el 65,4% considera que estas vacunas son seguras. En conclusión, la mayoría de los adultos mayores presenta un alto nivel de conocimientos, actitudes y prácticas y una percepción positiva sobre la vacuna contra la COVID-19.

## INTRODUCCIÓN

A inicios del 2022, la Organización Mundial de la Salud (OMS) reportó aproximadamente 300 millones de casos de COVID-19, más de cinco millones de muertes, y nueve mil millones de vacunas administradas ^1^. La COVID-19 ha afectado desproporcionadamente a los adultos mayores, evidenciando su vulnerabilidad, así como la fragilidad de los sistemas de salud. Los adultos mayores son más propensos a desarrollar formas severas de la enfermedad por cambios en su inmunidad y por tener enfermedades crónicas, lo que genera altas tasas de hospitalización y mortalidad ^2^. En el 2021, Perú reportó la mayor tasa de mortalidad per cápita por COVID-19 a nivel mundial, ello atribuido a la fragmentación del sistema de salud y a la escasez de recursos hospitalarios ^3^ ^,^ ^4^. En el Perú, los adultos mayores representan el 13% de la población nacional ^5^ y el 70% de defunciones por la COVID-19 ^6^. 

La vacunación es la medida más apropiada para controlar la infección ante la falta de intervenciones terapéuticas eficaces. El proceso de vacunación contra la COVID-19 en el Perú inició en febrero del 2021, y para el 28 de mayo del 2022 se habían aplicado más de 70 millones de dosis ^7^. Durante el 2021, los adultos mayores fueron inmunizados con la vacuna de Pfizer y en algunas regiones con AstraZeneca. El retraso en la vacunación podría propagar variantes que superen la inmunidad conferida por vacunas previas o por la enfermedad ^8^. Pese a ello, la baja aceptación de las vacunas es un problema que impide mitigar el padecimiento. Según encuestas realizadas en Perú, la aceptación de la vacuna contra la COVID-19 alcanzó un 49 a 60% entre enero y marzo del 2021 ^9^. 

El nivel de conocimiento sobre la COVID-19 repercute sobre la adopción de medidas preventivas, por lo que es necesario elaborar intervenciones educativas en poblaciones vulnerables ^10^. Mundialmente, se han realizado estudios para medir los conocimiento, actitudes y prácticas en diversas enfermedades a través del formato KAP (*Knowledge, Attitude and Practices*, por sus siglas en inglés); sin embargo, pocos trabajos se han realizado en adultos mayores ^11^ ^,^ ^12^. Dada la falta de evidencia en Perú y considerando la vulnerabilidad de los adultos mayores en la actual pandemia, el objetivo del presente estudio fue describir la percepción sobre las vacunas y el nivel de conocimientos, actitudes y prácticas relacionadas con la COVID-19, en adultos mayores que acuden a un hospital general de Lima, Perú.

**MENSAJE CLAVE**

Motivación para realizar el estudio: la percepción y el nivel de conocimientos, actitudes y prácticas hacia la COVID-19 influyen en la adopción de conductas preventivas en la sociedad, lo cual es importante en adultos mayores dada su vulnerabilidad epidemiológica.

Principales hallazgos: la mayoría de los adultos mayores presentó un alto grado de aceptación hacia las vacunas contra la COVID-19 y adecuado nivel de conocimientos, actitudes y prácticas.

Implicancias: es importante educar a poblaciones vulnerables sobre la COVID-19 para prevenir eventos adversos en su salud. Dada la heterogeneidad sociocultural del Perú, se requieren estudios en diferentes regiones del país para poder realizar intervenciones educativas específicas.

## EL ESTUDIO

### Diseño del estudio

Se realizó un estudio descriptivo, de corte transversal. 

### Población

La población estuvo conformada por 320 adultos mayores de 60 años a más que acudieron al consultorio de Valoración Geriátrica Integral (VGI) del Hospital Cayetano Heredia desde enero del 2019 a febrero del 2020. Se incluyeron adultos mayores de 60 años a más, que acudieron al consultorio de VGI y que tuvieron un teléfono móvil propio o de algún familiar registrado en la base de datos del consultorio de VGI. Se excluyeron pacientes quechuahablantes, con deterioro cognitivo, hipoacusia severa o trastorno del lenguaje. Se consideró al total de la población. Los pacientes fueron evaluados en el área física, cognitiva, afectiva, social, y otorgaron su consentimiento informado para evaluaciones telefónicas posteriores. 

### Instrumento

Para evaluar los conocimientos, actitudes y prácticas hacia la COVID-19, se empleó un instrumento en formato KAP basado en el estudio de Erfani et al., que fue elaborado originalmente por la OMS ^13^. Este instrumento fue aplicado en Irán en mayores de 15 años. Contiene 53 preguntas de opción múltiple. Dado que está escrito en inglés se realizó una validación cultural. 

### Validación cultural

Para validar el instrumento KAP se empleó el método de traducción-retraducción a cargo de tres médicos bilingües hispanohablantes nativos con un nivel de inglés avanzado. Este método consiste en traducir el instrumento al idioma de interés y posteriormente retrotraducirlo al idioma original ^14^. Se buscaron coincidencias simples entre las frases traducidas y se convirtieron en un porcentaje promedio. Luego, se halló la correlación del porcentaje de frases coincidentes entre las traducciones, calculando un promedio final. Finalmente, se evaluó la concordancia conceptual entre los ítems mediante lectura simple. Se realizaron los mismos pasos para evaluar las retrotraducciones. El proceso de validación cultural se describe en el material suplementario (S1 y S2). 

La versión traducida y retrotraducida del instrumento KAP fue entregada a un comité conformado por dos médicos generales y un médico internista, quienes elaboraron un documento de consenso (S3 y S4). Con esta versión validada, se realizó una prueba piloto en 58 adultos mayores de la comunidad a través de redes sociales (S5 y S6). El instrumento KAP final incluyó 54 preguntas (S7). La percepción sobre las vacunas para la COVID-19 se evaluó con un cuestionario exploratorio elaborado por los investigadores (S8). 

### Recolección de datos

La encuesta fue realizada por vía telefónica del 24 de marzo al 14 de noviembre del 2021. La encuesta duró 20 min, las respuestas fueron registradas en fichas de recolección de datos y digitadas en Microsoft Excel 2016. 

Las variables recolectadas incluyeron características sociodemográficas (edad, sexo, estado civil, años de educación) y antecedentes médicos (comorbilidades y diagnóstico de COVID-19 durante la pandemia).

Las variables acerca de la percepción sobre las vacunas contra la COVID-19 incluyeron el antecedente de vacunación, número de dosis, percepción de seguridad de las vacunas, conocimiento sobre sus beneficios, relevancia de la vacunación, y el conocimiento de medidas preventivas luego de ser vacunado. 

Las variables del instrumento KAP se dividieron en: 

Conocimientos: evaluó la etiología, características, forma de presentación y manejo de la enfermedad. 

Actitudes: evaluó percepción de gravedad de la enfermedad, la cuarentena como medida preventiva, vías de transmisión de la enfermedad y aceptación de medidas gubernamentales.

Prácticas: evaluó medidas de aislamiento y prevención de contacto con posibles infectados. Así como la higiene personal, percepción de medicina homeopática y fuente de información.

Análisis estadístico 

Se empleó el *software* STATA versión 16. Las variables categóricas se presentan como frecuencias y proporciones, las variables continúas se presentan con la media y desviación estándar (DE) o con la mediana y rangos intercuartílicos (RIC), de acuerdo con su distribución normal medida con la prueba de Shapiro-Wilk.

Aspectos éticos

Se respetaron los principios éticos delineados en la Declaración de Helsinki. El estudio fue aprobado por el Comité de Ética del Hospital Cayetano Heredia (CIE-HCH), con código 124-2020.

## HALLAZGOS

Se contactaron telefónicamente a 320 adultos mayores, de los cuales 220 no contestaron la llamada, quedando 100 que aceptaron participar en el estudio. De ellos, 17 fueron excluidos por no cumplir con los criterios de inclusión. La muestra final fueron 83 participantes, siendo 62,7% mujeres. La edad media fue de 74,0 (DE: 7,7) años, y el estado civil más frecuente fue el casado (46,9%). El 25,3% de los participantes refirió haber tenido la COVID-19, durante la pandemia (Tabla 1).

**Tabla 1:** Características sociodemográficas de los adultos mayores del estudio (n=83)

Variables	n (%)
Edad ^a^	74,0 (7,7)
Sexo	
Femenino	52 (62,7)
Masculino	31 (37,3)
Estado civil	
Soltero	20 (24,1)
Casado	39 (47,0)
Viudo	16 (19,3)
Divorciado	5 (6,1)
Conviviente	3 (3,6)
Años de educación	
0 años	9 (10,8)
1-6 años	39 (47,1)
7-11 años	27 (32,5)
12 años a más	8 (9,6)
Diagnóstico de COVID-19 durante la pandemia	21 (25,3)
Antecedentes	
Hipertensión arterial	37 (44,6)
Diabetes mellitus	18 (21,7)
Falla cardiaca	1 (1,2)
Enfermedad renal crónica	4 (4,8)
Arritmia	3 (3,6)
Asma bronquial	9 (10,8)
EPOC	2 (2,4)
Hipotiroidismo	6 (7,2)
Otros	3 (3,7)

EPOC: enfermedad pulmonar obstructiva crónica

a Media (DE)

Sobre la percepción de las vacunas, el 91,5% de los participantes recibió al menos una dosis. El 95% reportó que las vacunas protegen de formas severas de enfermedad, el 9,9% indicó que no son segura (Tabla 2). Respecto a los conocimientos, el 98,8% ha escuchado sobre la enfermedad, el 92,7% sabe que es contagiosa y el 73,4% conoce su etiología viral. El 92,7% desconoce el período de incubación y el 50,6% cree que puede transmitirse por lácteos o carne contaminada. El 43,3% refirió que no hay tratamiento específico o solo es paracetamol, y el 22,8% indicó que es ivermectina (Tabla 3). En las actitudes, el 96,3% considera que es una enfermedad grave y el 65,4% refirió que el nivel de conciencia social sobre la enfermedad es insuficiente (Tabla 4). En cuanto a las prácticas, el 88,8% evita salir de casa para prevenir el contagio, el 93,8% evita el contacto físico, el 100% le presta más atención a su higiene y el 61,7% usa medicina tradicional para prevenir la enfermedad (Tablas 5).

**Tabla 2:** Percepción sobre las vacunas contra la COVID-19 en adultos mayores

Ítem	n (%)
1. ¿Se ha puesto la vacuna para la influenza o neumococo? (n=81/83) ^a^	
No sabe	3 (3,7)
No	20 (24,7)
Sí	58 (71,6)
2. Si la respuesta fue afirmativa, ¿cuál vacuna se ha administrado? (n=58)	
Neumococo	13 (22,4)
Influenza	9 (15,5)
Ambos	36 (62,1)
3. ¿Se vacunó contra la COVID-19? (n=71/83) ^a^	
Sí	65 (91,5)
No	6 (8,5)
4. Si la respuesta fue afirmativa, indicar número de dosis para COVID-19 (n=64/65) ^a^	
1ra dosis	6 (9,4)
2da dosis	58 (90,6)
5. ¿Considera que es la misma vacuna contra la influenza? (n=81/83) ^a^	
No sabe	15 (18,5)
No	60 (74,1)
Sí	6 (7,4)
6. ¿Cree que una persona no vacunada contra COVID-19 probablemente contraerá COVID-19 y que podría complicarse, es decir hospitalizarse y/ o morir? (n=80/83) ^a^	
No sabe	7 (8,8)
No	1 (1,2)
Sí	72 (90,0)
7. ¿Considera que las vacunas son seguras? (n=81/83)^ a^	
No sabe	20 (24,7)
No	8 (9,9)
Sí	53 (65,4)
8. ¿Cree que las vacunas pueden evitar formas severas de enfermedad? (n=80/83) ^a^	
No sabe	3 (3,8)
No	1 (1,2)
Sí	76 (95,0)

a Datos perdidos

**Tabla 3:** Conocimientos sobre COVID-19 en adultos mayores.

Ítem	n (%)
K1. He escuchado acerca del COVID-19 (respuesta: sí)	82 (98,8)
K2. La COVID-19 es una enfermedad contagiosa (respuesta: sí)	77 (92,7)
K3. ¿Cuál de las siguientes es causa de la COVID-19? (respuesta: virus)	61 (73,4)
K4. ¿Cuál es la duración del período de incubación de la enfermedad? (respuesta: no sabe)	77 (92,7)
K5. ¿Cuál de los siguientes es el tratamiento para la COVID-19? (respuesta: no hay tratamiento/paracetamol) ^a^	36 (43,3)
K6. ¿En cuál grupo de edades la enfermedad es más peligrosa? (respuesta mayores de 50 años) ^b^	38 (45,7)
K7. La fiebre es un síntoma de la COVID-19 (respuesta: sí)	67 (80,7)
K8. La tos es un síntoma de la COVID-19 (respuesta: sí)	65 (92,7)
K9. El dolor de garganta es un síntoma de la COVID-19 (respuesta: sí)	71 (85,5)
K10. El dolor de cuerpo es un síntoma de la COVID-19 (respuesta: sí)	72 (86,7)
K11. La diarrea o el estreñimiento son un síntoma de la COVID-19 (respuesta: sí)	66 (79,5)
K12. El dolor de cabeza es un síntoma de la COVID-19 (respuesta: sí)	70 (84,3)
K13. Si sospecho de una infección por COVID-19, antes que todo, mido la fiebre (respuesta: sí)	69 (84,1)
K14. Si sospecho de una infección por COVID-19, antes que todo, busco a un médico (respuesta: sí)	72 (87,8)
K15. Si sospecho de una infección por COVID-19, evito realizar actividades diarias innecesarias (respuesta: sí)	73 (89,0)
K16. Para evitar contraer el COVID-19, yo evito el contacto con individuos con sospecha de estar infectados por COVID-19 (respuesta: sí)	78 (95,1)
K17. El número de casos de la enfermedad están aumentando en el Perú (respuesta: sí)	44 (54,3)
K18. Lavarse las manos con agua y jabón puede eliminar la causa de la enfermedad (respuesta: sí)	75 (92,5)
K19. La enfermedad puede ser transmitida directamente mediante la tos (respuesta: sí)	72 (88,8)
K20. La enfermedad puede ser transmitida directamente mediante el contacto con superficies infectados (respuesta: sí)	64 (79,0)
K21. La enfermedad puede ser transmitida directamente mediante el consumo de lácteos y carne contaminados (respuesta: sí)	41 (50,6)
K22. La enfermedad puede ser transmitida directamente mediante el contacto con individuos infectados (respuesta: sí)	77 (95,0)
K23. La enfermedad es más peligrosa en mujeres gestantes (respuesta: sí)	58 (71,6)
K24. La enfermedad es más peligrosa en individuos mayores (respuesta: sí)	78 (96,3)
K25. La enfermedad es más peligrosa en personas con sistemas inmunes debilitados (respuesta: sí)	78 (96,3)
K26. La enfermedad es más peligrosa en personas con cáncer, diabetes y enfermedades respiratorias crónicas (respuesta: sí)	77 (95,0)

a Alternativas: a) Paracetamol / No hay tratamiento, b) Ivermectina, c) Antibióticos, d) Otros (anticoagulantes, corticoides, hierbas), e) No sabe

b Alternativas: a) Menores de 15 años, b) 15 a 30 años, c) 30 a 50 años, d) Más de 50 años, e) En todas las edades, f) No sabe

**Tabla 4:** Actitudes sobre COVID-19 en adultos mayores.

Ítem	n (%)
A1. Usted cree que la detección temprana de la COVID-19 puede mejorar el tratamiento y el desenlace final (respuesta: sí)	73 (90,1)
A2. Yo opino que la COVID-19 puede ser tratado en casa (respuesta: sí)	69 (85,1)
A3. Yo opino que la educación en salud puede prevenir la COVID-19 (respuesta: sí)	79 (97,5)
A4. Yo opino que la COVID-19 es una enfermedad grave (respuesta: sí)	78 (96,3)
A5. Yo opino que la COVID-19 puede ser prevenida mediante una adecuada cuarentena (respuesta: sí)	71 (87,6)
A6. Yo opino que, si existe una vacuna disponible para la enfermedad, debe de usarse (respuesta: sí)	77 (95,0)
A7. Yo opino que la COVID-19 es una enfermedad curable (respuesta: sí)	66 (81,4)
A8. Yo opino que es suficiente el nivel de conciencia de la sociedad sobre la COVID-19 (respuesta: no)	53 (65,4)
A9. Yo opino que la COVID-19 produce la muerte en todos los casos (respuesta: no)	57 (70,3)
A10. Yo opino que la COVID-19 puede ser transmitido a los humanos a través de las mascotas (respuesta: no)	34 (41,9)
A11. Yo opino que las autoridades deben restringir los viajes desde y hacia las áreas con COVID-19 para prevenir la contaminación (respuesta: sí)	74 (91,3)
A12. Yo opino que las autoridades deben poner en cuarentena a los pacientes con COVID-19 en hospitales especiales (respuesta: sí)	69 (85,1)
A13. Yo opino que por el incremento en el número de casos de COVID-19 han hecho bien en cerrar los centros educativos (respuesta: sí)	77 (95,0)
A14. Yo opino que por el incremento en el número de casos de la enfermedad COVID-19 han hecho bien en cerrar lugares religiosos como las iglesias (respuesta: sí)	68 (83,9)
A15. Yo opino que por el incremento del número de casos de COVID-19 las autoridades deben estar listas para cerrar y poner nuevamente en cuarentena a la ciudad (respuesta: sí)	54 (66,6)

**Tabla 5:** Prácticas sobre la COVID-19 en adultos mayores.

Ítem	n (%)
P1. Con la intención de prevenir el contagio y la diseminación de la COVID-19 yo evito salir de mi casa (respuesta: sí)	72 (88,8)
P2. Con la intención de prevenir el contagio y la diseminación de la COVID-19, yo evito tomar viajes innecesarios (respuesta: sí)	76 (93,8)
P3. Con la intención de prevenir el contagio y la diseminación de la COVID-19 yo evito comer comida fuera de casa (respuesta: sí)	79 (97,5)
P4. Con la intención de prevenir el contagio y la diseminación de la COVID-19, yo evito saludar con la mano, abrazar y besar (respuesta: sí)	76 (93,8)
P5. Con la intención de prevenir el contagio y la diseminación de la COVID-19, yo me lavo las manos frecuentemente (respuesta: sí)	81 (100,0)
P6. Con la intención de prevenir el contagio y la diseminación de la COVID-19, yo le presto más atención que la usual a mi higiene personal (respuesta: sí)	81 (100,0)
P7. Con la intención de prevenir el contagio y la diseminación de la COVID-19, yo uso desinfectantes y soluciones (lejía, alcohol en gel) (respuesta: sí)	75 (92,5)
P8. Con la intención de prevenir el contagio de la COVID-19, yo utilizo productos a base de hierbas y medicina tradicional (respuesta: sí)	50 (61,7)
P9. Con la intención de prevenir el contagio de la COVID-19, yo tomo suplementos vitamínicos (respuesta: no)	54 (66,6)
P10. Con la intención de prevenir el contagio y la diseminación de la COVID-19, ¿Cuándo usa máscaras faciales? (respuesta: lugares públicos) ^a^	40 (49,3)
P11. Con la intención de prevenir el contagio de la COVID-19, ¿usted piensa que (las gárgaras, el eucalipto, los ajos o el kion) pueden prevenirlo? (respuesta: sí)	50 (62,5)
P12. Con la intención de curar o tratar la COVID-19 ¿usted piensa que (las gárgaras, el eucalipto, los ajos o el kion) pueden curarlo? (respuesta: no)	43 (53,7)
P13. ¿Cuál es su principal fuente de información sobre el conocimiento de la enfermedad? (respuesta: televisión) ^b^	29 (35,8)

a Alternativas: a) Nunca, b) Lugares públicos y llenos de gente, c) La mayoría del tiempo, d) Siempre, e) No sabe

b Alternativas: a) Televisión, b) Radio, c) Periódicos, d) Redes sociales, e) Personal de salud, f) Familia y amigos, g) Vecinos

## DISCUSIÓN

En el presente estudio, la mayoría de los adultos mayores reconocieron a las vacunas como una medida preventiva, y además presentaron un adecuado nivel de conocimiento, actitudes y prácticas hacia la COVID-19. Sin embargo, también se encontraron creencias erróneas que podrían influir en sus conductas preventivas frente a la pandemia. 

Respecto a la percepción sobre las vacunas contra la COVID-19, la gran mayoría de los participantes reportó haber sido vacunado y recibido dos dosis, lo cual es similar a la situación epidemiológica local durante el período de estudio (96,6% de adultos mayores vacunados con segunda dosis en Lima Norte y 90% a nivel nacional) ^7^. Estos hallazgos sugieren que la aceptación de las vacunas en este grupo etario es alta. Esto contrasta con un estudio realizado a inicios de la implementación de vacunas en el Perú, donde la intención de vacunación en adultos mayores fue del 64,4% ^15^. La diferencia observada podría deberse a las campañas de vacunación masivas realizadas a nivel nacional, sin embargo, la vacunación también pudo haber sido influenciada por decisión de los familiares del adulto mayor. 

Sobre los conocimientos, casi todos los participantes conocen el origen viral y los síntomas. Esto podría atribuirse a que el Ministerio de Salud promovió la difusión de información por medios de comunicación desde inicios de la pandemia. Estos hallazgos difieren de estudios internacionales en los que el nivel de conocimientos está inversamente relacionado con la edad ^12^ ^,^ ^16^. Por otro lado, los participantes reportaron creencias erróneas sobre la historia natural de la enfermedad, como la posibilidad de transmisión por alimentos. Además, la mayoría desconocía el período de incubación, lo que sugiere que quizás ignoraron el tiempo de aislamiento requerido luego de la exposición a un contacto o a una persona con diagnóstico de COVID-19. Respecto al tratamiento, las respuestas no fueron uniformes, lo cual podría explicarse por las cambiantes medidas adoptadas para afrontar la pandemia en el Perú. Por ejemplo, el Estado promovió el uso de ivermectina, hidroxicloroquina y azitromicina al inicio de la pandemia, lo cual podría explicar la heterogeneidad de las respuestas. 

Otro hallazgo relevante fue que casi la mitad de los participantes consideró que la enfermedad es más peligrosa en adultos mayores de 50 años; una proporción similar manifestó que afecta por igual a todas las edades. Esto indicaría que una parte importante infravalora el riesgo de los adultos mayores para tener formas severas de la COVID-19. Sin embargo, es importante considerar que durante el período de estudio aumentaron los casos en adultos jóvenes en nuestro país, lo cual podría haber influido en la perspectiva de los participantes. Varios estudios internacionales han evidenciado que la percepción de riesgo de los adultos mayores frente a la COVID-19 está subestimada, lo cual es atribuido a un fenómeno de optimismo ilusorio paradójico ^17^. 

En cuanto a las actitudes, la mayoría de los adultos mayores reconoció que la COVID-19 es una enfermedad grave, y que la educación en salud y la cuarentena pueden prevenir su transmisión. Un hallazgo notable en este dominio fue que más de la mitad considera que la sociedad no tiene suficiente nivel de conciencia sobre el impacto de la COVID-19. Sobre las prácticas frente a esta enfermedad, la mayoría adoptó medidas de bioseguridad para evitar el contagio, tal como se ha descrito en otros estudios ^12^ ^,^ ^17^. Además, más de la mitad usaron productos a base de hierbas y medicina tradicional para prevenir el contagio. Similarmente, un estudio en población peruana infectada con VIH halló que casi la mitad de los participantes (46,8%) creía que las gárgaras con agua y sal previenen la COVID-19 ^18^. Otro estudio en Cusco indicó que el 80,2% usaron plantas medicinales para prevenir, mientras que el 71% para tratar la COVID-19 ^19^.

Este estudio tiene la fortaleza de ser uno de los primeros en indagar la percepción de los adultos mayores sobre las vacunas contra la COVID-19 y sobre la pandemia en el Perú. Las principales limitaciones fueron un tamaño de muestra reducido debido al limitado uso de telefonía móvil en el adulto mayor. En este estudio, solamente el 10% de los encuestados tenía un celular propio, lo que puede deberse a los bajos recursos económicos. Además, el cambio constante de los números de celular en la familia puede haber limitado el contacto con el adulto mayor, esto explicaría la pobre tasa de respuesta del estudio.

Por otro lado, durante el período de estudio el número de casos de COVID-19 fue en descenso debido a la vacunación, lo que se advierte por la heterogeneidad en las respuestas de los participantes. Por ejemplo, a mediados de abril del 2021, se producían alrededor de 1600 casos diarios, mientras que en septiembre del mismo año se presentaban alrededor de 1000 casos diarios ^20^. Además, el uso de la vía telefónica no fue la adecuada para la recolección de datos, dada la longitud de la encuesta empleada. Asimismo, las respuestas de los participantes pueden haber estado influenciadas por familiares que estuvieron presentes durante la llamada. Debido a que los participantes fueron todos procedentes de la ciudad de Lima, no se pueden extrapolar los resultados a otras regiones del Perú. Asimismo, la presente validación cultural no es aplicable en poblaciones con lenguas maternas distintas al español. 

En conclusión, la mayoría de los adultos mayores encuestados presentan una percepción positiva sobre la vacuna contra la COVID-19 y un nivel de conocimientos, actitudes y prácticas aceptables hacia esta enfermedad. Sin embargo, también presentaron numerosas creencias erróneas que podrían repercutir sobre las prácticas preventivas. Se requiere elaborar más estudios en adultos mayores para obtener evidencia sobre sus percepciones y de esta forma implementar estrategias sanitarias y educativas frente a posibles futuras pandemias.
